# 3C methods in cancer research: recent advances and future prospects

**DOI:** 10.1038/s12276-024-01236-9

**Published:** 2024-04-25

**Authors:** Insoo Yoon, Uijin Kim, Kyung Oh Jung, Yousuk Song, Taesoo Park, Dong-Sung Lee

**Affiliations:** 1 Department of Life Science, University of Seoul, Seoul, 02504 Republic of Korea; 2 Department of Anatomy, College of Medicine, Chung-Ang University, Seoul, 06974 Republic of Korea

**Keywords:** Cancer genomics, Epigenomics

## Abstract

In recent years, Hi-C technology has revolutionized cancer research by elucidating the mystery of three-dimensional chromatin organization and its role in gene regulation. This paper explored the impact of Hi-C advancements on cancer research by delving into high-resolution techniques, such as chromatin loops, structural variants, haplotype phasing, and extrachromosomal DNA (ecDNA). Distant regulatory elements interact with their target genes through chromatin loops. Structural variants contribute to the development and progression of cancer. Haplotype phasing is crucial for understanding allele-specific genomic rearrangements and somatic clonal evolution in cancer. The role of ecDNA in driving oncogene amplification and drug resistance in cancer cells has also been revealed. These innovations offer a deeper understanding of cancer biology and the potential for personalized therapies. Despite these advancements, challenges, such as the accurate mapping of repetitive sequences and precise identification of structural variants, persist. Integrating Hi-C with multiomics data is key to overcoming these challenges and comprehensively understanding complex cancer genomes. Thus, Hi-C is a powerful tool for guiding precision medicine in cancer research and treatment.

## Hi-C technology unlocks cancer mysteries: insights for precision medicine

The research explores the 3D structure of chromatin—a combination of DNA and proteins in our cells, and its role in cancer. Using Hi-C technology, a method that maps the closeness of different DNA regions within the cell nucleus, researchers have gained insights into these 3D structures, especially in cancer cells. The study involves data analysis using Hi-C to investigate chromatin’s spatial arrangement and its implications for cancer. By studying interactions between various DNA regions, researchers identify crucial chromatin structures and understand how changes in these structures contribute to cancer. The findings reveal that disruptions in chromatin structure, such as altered interactions between enhancer, promoter, and the formation of additional DNA, play a significant role in cancer development. These insights could lead to new diagnostic and treatment strategies, focusing on targeting the 3D chromatin structure in cancer cells.

This summary was initially drafted using artificial intelligence, then revised and fact-checked by the author.

## Introduction

Three-dimensional (3D) chromatin organization within the nucleus is a dynamic and intricate phenomenon that substantially influences various cellular processes, including gene regulation, DNA replication, and development^1^. Understanding the spatial arrangement and interactions of genomic elements is crucial for revealing the complexities of diseases, with cancer being a prominent focus of contemporary research^2–4^. Over the past decade, the emergence of chromatin conformation capture (3C) techniques and the subsequent development of Hi-C technology have enhanced our ability to dissect the 3D chromatin landscape, offering unparalleled insights into genomic structure and function^5,6^. In this study, we explore recent advancements in Hi-C technology, shedding light on its applications in deciphering the multifaceted realm of 3D chromatin interactions, with a primary emphasis on cancer research.

The cornerstone of Hi-C methodology lies in its ability to capture and quantify the spatial proximity of genomic regions within the nucleus. This powerful approach enables the comprehensive study of critical chromatin structures, including chromatin loops, topologically associating domains (TADs), and the roles of architectural proteins, such as CCCTC-binding factor (CTCF) and cohesin, in shaping these structures^7–9^. Moreover, the application of Hi-C can extend beyond primary chromatin structures to explore higher-order chromatin organization, thereby providing a holistic view of how the genome functions as an integrated system.

Advanced tools that can delve into the genetic intricacies of malignant transformation can immensely benefit the study of cancer, which is characterized by genomic instability and aberrant gene regulation^3^. Recent advancements in Hi-C are revolutionizing cancer research by elucidating the roles of oncogene activation, structural variations (SVs), haplotype phasing, and extrachromosomal DNA (ecDNA) in the occurrence and progression of cancer^10–13^.

This paper aims to reveal the importance of these cutting-edge Hi-C technologies and their specific contributions to our understanding of cancer biology. From the precise identification of structural variants that activate oncogenes to the elucidation of allelic variations at the haplotype level, Hi-C is a powerful tool for studying the often elusive mechanisms of cancer initiation and progression. Notably, the detection of ecDNA and its effects on gene regulation and amplification in cancer adds another dimension to our understanding of this complex disease.

However, it is essential to acknowledge that Hi-C research is challenging. The existence of repetitive sequences and the need for optimized methods to map SVs are some of the hurdles that researchers must overcome. Nevertheless, the integration of Hi-C technology with multiomics datasets can provide a holistic and multidimensional view of cancer genomes, fostering a deeper understanding that could shape future cancer research and clinical applications. This paper highlights the remarkable potential of Hi-C in revealing the dynamic 3D chromatin architecture of cancer cells.

## Principle and analysis of the Hi-C sequencing method

**Fig. 1:**
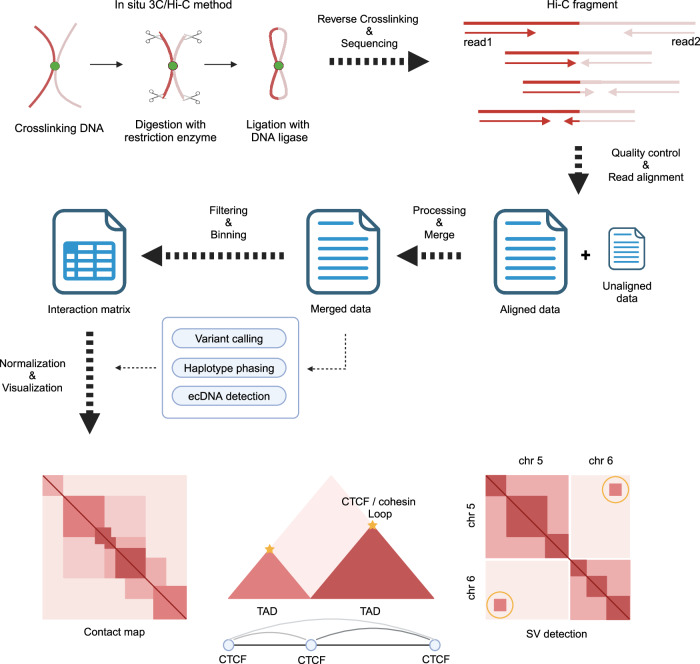
Overview of the Hi-C sequencing method and analysis pipeline. In situ Hi-C is performed through DNA crosslinking, digestion, and ligation steps. A Hi-C sequencing library is constructed, and paired-end sequencing reads are generated via high-throughput sequencing platforms. The raw data are aligned to the reference genome after quality control. Unaligned reads that can remain because of the biased Hi-C sequencing library are processed and merged with the aligned data. Further processes, such as variant calling, haplotype phasing, and ecDNA detection, can be performed using the merged aligned data with various bioinformatics computational tools. This information can be integrated into an interaction matrix containing contact frequencies between genomic regions to create a contact map, topologically associated domains (TADs), and structural variant (SV) information.

Within mammalian cells, DNA is organized into nucleosomes and then assembles into more complex chromatin structures, which play important roles in controlling the cell cycle, replication, development, and gene activity. Various interactions take place within these 3D chromatin structures, including those that boost gene expression, such as enhancer–promoter interactions, and those that repress gene expression, such as interactions with lamina-associated domains. The organization of the chromatin structure relies on key structural components, such as CTCF and cohesion^1,7^. The 3C technique can be used to map interactions between particular genomic regions^5^. Although 3C is effective for assessing changes in the spatial proximity of two regions under different conditions, it has limitations in providing a comprehensive understanding of the entire 3D structure beyond the specific regions being examined. Research has been conducted to solve this problem, and Hi-C, a relatively new technology, has enabled the profiling of the entire genome structure^6^ (Fig. 1). The procedure commences with cell fixation to maintain the spatial arrangement of the chromatin. This step is followed by DNA cleavage using restriction enzymes. The subsequent ligation step enhances the chances of connecting DNA fragments located close to each other. Subsequently, the joined DNA sequences are separated and used to construct a sequencing library. The prepared Hi-C library is sequenced using a high-throughput sequencing platform. The raw sequencing reads are subjected to quality control to remove low-quality reads and adapter sequences. The reads are aligned to the reference genome using specialized alignment algorithms to determine their genomic locations. Chimeric reads can also be generated from the Hi-C fragments. The unaligned reads undergo further processing and are integrated into the aligned data. The aligned data are analyzed to identify pairs of genomic loci that were in close proximity to the original sample. Contact frequencies between pairs of loci are calculated, representing the frequency of interactions between genomic regions, and the information is stored in an interaction matrix. The following operations are performed only on the aligned data: variant calling, haplotype phasing, and ecDNA detection. Contact frequencies are normalized to correct biases introduced during library preparation and sequencing, considering factors such as genomic distance or mappability. Normalized data are visualized as contact maps, where each cell represents the interaction frequency between two genomic loci. Various bioinformatic tools and algorithms are used to analyze contact maps and identify chromatin loops, TADs, compartments, SVs, and other higher-order chromatin structures.

## Progress in the development of 3D genome interaction research

In recent years, considerable progress has been made in 3D genome analysis techniques, resulting in an influx of data. However, the majority of currently available Hi-C data offer resolutions between 25 kb and 1 Mb. High-resolution Hi-C data, typically ranging from 1 to 10 kb, are only accessible for a limited number of tissues or cell lines, thus affecting our ability to examine structures at the kb pair level. Notably, as data resolution increases, so does the need for more extensive sequencing, and consequently, the associated costs increase.

Recently, several computational techniques have been developed to improve the resolution of Hi-C data. For example, HiCPlus uses a superresolution convolutional neural network algorithm to replace the original matrix with a very similar interaction matrix using only 1/16 of the original sequencing reads^14^. HiCNN can efficiently enhance low-resolution Hi-C data using a 54-layer, very deep convolutional neural network to enhance the resolution of Hi-C data^15^. Li et al.^16^ developed SRHiC, a deep learning-based computational method, and inferred the corresponding high-resolution Hi-C interaction matrix from a low-resolution matrix with fewer layers. HiDENSEC, a new computational framework for analyzing heterogeneous solid cancers, was developed to predict copy number alterations, large-scale chromosomal rearrangements, and cancer cell fractions^17^.

In addition, considerable efforts have been made to improve the specificity of chromatin conformation capture techniques (Table 1). Chromatin immunoprecipitation-loop assay (ChIP-loop), a method that combines ChIP and 3C technology to identify protein-mediated chromatin interactions, has been developed^18^. Fullwood et al.^19^ implemented an enrichment strategy using a more extended method, chromatin interaction analysis with paired-end tags (ChIA-PET), and were able to identify chromatin associations mediated by specific proteins of interest at a genome-wide scale without preference^20^. Proximity ligation-assisted ChIP-seq (PLAC-seq)^21^ and in situ Hi-C followed by chromatin immunoprecipitation (HiChIP)^22^ improves the DNA contact capture efficiency and accuracy by applying in situ Hi-C, and HiChIP uses a Tn5 transposase-mediated sequencing library construction approach. ChIA-drop can isolate single chromatin complexes without ligation via droplet-based and barcode-linked sequencing^23^.

**Table 1:** Methods for confirming chromatin conformations and the types of data they provide.

Bulk-cell methods	Data type 1	Data type 2	Data access (GEO)	Ref.
Hi-C	Chromatin conformation		GSE18199	^6^
ChIA-PET	Chromatin conformation		GSE18046	^19,20^
PLAC-seq	Chromatin conformation		GSE86150	^21^
HiChIP	Chromatin conformation		GSE80820	^22^
ChIA-drop	Chromatin conformation		GSE109355	^23^
**Single-cell methods**				
scHi-C	Chromatin conformation		GSE48262	^24^
snHi-C	Chromatin conformation		GSE94489	^25^
scMethyl-HiC	Chromatin conformation	DNA methylome	GSE119171	^28^
sn-m3C-seq	Chromatin conformation	DNA methylome	GSE124391 GSE130711	^29^
HiRES	Chromatin conformation	Transcriptome	GSE223917	^30^
scCARE-seq	Chromatin conformation	Transcriptome	GSE211395	^31^
LiMCA	Chromatin conformation	Transcriptome	GSE239969	^32^

*ChIA-Drop* chromatin interaction analysis via droplet-based and barcode-linked sequencing, *ChIA-PET* chromatin interaction analysis with paired-end tags, *Hi-C* high-throughput chromosome conformation capture, *HiChIP* Hi-C followed by chromatin immunoprecipitation, *HiRES* Hi-C and RNA-seq employed simultaneously, *LiMCA* linking mRNA to chromatin architecture, *PLAC-seq* proximity ligation-assisted ChIP-seq, *scCARE-seq* simultaneous detection of chromatin architecture and messenger RNA expression by sequencing, *scHi-C* single-cell Hi-C, *scMethyl-HiC* single-cell Methyl-HiC, *snHi-C* single-nucleus Hi-C, *sn-m3c-seq* single-nucleus methyl-3C sequencing.

## Development of single-cell techniques for profiling chromatin structure

The initial single-cell Hi-C (scHi-C) analysis was performed on mouse CD4+ T cells and involved a sequential process of chromatin crosslinking, restriction enzyme digestion, biotin filling, and ligation within the permeated nuclei^24^. Subsequently, numerous single nuclei were manually isolated after ligation, and biotinylated ligation junctions were individually pulled down from each isolated nucleus. Using single-cell Hi-C technology, Nagano et al.^25^ reported that mouse embryonic stem cells undergo chromosomal condensation during the early G1 phase, followed by substantial reorganization during replication. This finding implies a correlation between the cell cycle and variations in chromatin interactions. Additionally, the adoption of single-nucleus Hi-C eliminated the conventional biotin-filling step instead of employing a sticky-end ligation method. This modification enabled the distinct reconstruction of the chromatin structure during the transition from oocytes to zygotes in mice^26^. Another method, Dip-C, also excludes biotin-related steps and has been applied to single human diploid cells (GM12878) to amplify DNA through transposon-based whole-genome amplification to obtain data on haplotypes^27^.

Recent advancements have led to the development of single-cell multiomic Hi-C techniques that allow simultaneous Hi-C and bisulfite sequencing from the same cell (Table 1). Single-cell methyl-HiC (scMethyl-HiC) was used to perform in situ Hi-C and bisulfite conversion^28^. Single-nucleus methyl-3C sequencing (sn-m3C-seq) was performed on a Hi-C instrument without the ligation junction enrichment step^29^. These techniques enable the identification of subcell populations based on known DNA methylation profiles as cell type-specific epigenetic signatures. In a recent study, a multiomics profiling technique that can efficiently and accurately determine both 3D genome structure and gene expression was developed (Table 1). Liu et al.^30^ used Hi-C and RNA-seq simultaneously (HiRES) to generate a 3D genome and transcriptome atlas of mouse embryos after implantation and identified the interaction of multiple factors that jointly shape the single-cell 3D genome during development. In addition, single-cell multimodal omics methods allowing the simultaneous detection of chromatin architecture and messenger RNA (mRNA) expression by sequencing (scCARE-seq)^31^ and linking mRNA to chromatin architecture (LiMCA)^32^ have been developed to coprofile chromatin architecture and transcriptomes. These studies identified changes in the processes that control the regulation of olfactory receptor expression and confirmed that periodic changes in chromatin structure and transcription are interconnected in the cell cycle. These single-cell multiomics Hi-C methods facilitate the concurrent characterization of cell type-specific chromatin organization and epigenomes in complex tissues or heterogeneous cancer genomes.

Considering the historical reliance on tumor heterogeneity signals to infer cancer traits, these innovative methodologies present invaluable resources for mapping the development of tumors. Gaining insights into the source of mutations in the course of tumorigenesis can reveal the mutations that promote tumor initiation, proliferation, infiltration, and metastasis. This knowledge is pivotal in advancing our understanding of cancer development and progression.

## Recent cancer studies from the perspective of chromatin structural levels

**Fig. 2:**
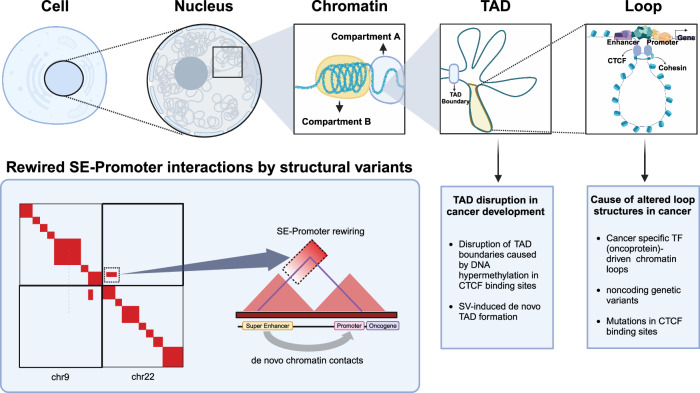
Deciphering the 3D genomic landscape by investigating chromatin loops and SV-induced tumorigenesis. Chromatin is a complex of DNA and proteins that is organized in the nucleus. It is divided into compartments A and B, which represent different structural and functional states, respectively. TADs are self-interacting genomic regions that play crucial roles in organizing chromatin into discrete functional units. Enhancers are regulatory DNA sequences that enhance the transcription of specific genes. These enhancers often interact with gene promoters over long distances, forming DNA loops that bring distant regulatory elements in close proximity to their target genes. Chromosome conformation capture (3C) maps are used to study these 3D interactions and provide insights into the spatial organization of chromosomes and the formation of TADs and DNA loops. SVs are alterations in genomic DNA sequences, including insertions, deletions, duplications, inversions, and translocations. These variants can be detected through 3C maps, revealing instances of enhancer hijacking, where SVs alter these interactions and dysregulate gene expression, thereby contributing to the development of diseases, such as cancer.

Recent advancements in cancer research have revealed an intricate relationship between 3D chromatin structure and transcriptional regulation in cancer. Here, we summarize recent cancer studies related to chromatin structure by referring to previous reviews by Deng et al.^3^ and Wang et al. ^4^ (Table 2 and Fig. 2).

**Table 2:** Recent cancer studies related to chromatin structure.

Year	Studies	3D genome feature	Type of cancer	Refs.
2024	Ijaz, J. et al.	Chromothripsis	Esophageal adenocarcinoma	^113^
2024	Achinger-Kawecka, J. et al.	Alteration of genome 3D structure associated with decitabine treatment	Breast cancer	^124^
2023	Kim, K. et al.	Enhancer hijacking	Colorectal cancer	^110^
2023	Erdmann-Pham, D. D. et al.	Somatic SVs	Melanoma	^17^
2023	Song, T. et al.	Somatic SVs	Adenocarcinoma	^58^
2023	Li, P. et al.	Alteration of genome 3D structure associated with oxaliplatin resistance	Colorectal cancer	^123^
2022	Mallard et al.	Chromothripsis	Leukemia	^55^
2022	Kim, T. et al.	CTCF	Breast cancer	^122^
2022	Du, Y. et al.	Somatic SVs	Pancreatic cancer	^59^
2021	Sunkel et al.	TF(ocoprotein)-driven chromatin loop	Rhabdomyosarcoma	^40^
2021	Boone et al.	TF(ocoprotein)-driven chromatin loop	Ewing sarcoma	^41^
2021	Iyyanki et al.	Altered chromatin loops associated with FOXA1 and GATA3	Bladder cancer	^49^
2021	Montefiori et al.	Enhancer hijacking	Leukemia	^54^
2021	Sungalee et al.	Enhancer hijacking	Lymphoma	^57^
2021	Alpsoy et al.	CTCF	Prostate cancer	^83^
2021	Wong et al.	CTCF	Breast cancer	^84^
2021	Sivapragasam et al.	CTCF	Melanoma	^85^
2021	Surdez et al.	STAG2 cohesin complex subunit	Ewing sarcoma	^97^
2021	Carico et al.	Hinge domain of the cohesin complex	Acute myeloid leukemia	^98^
2021	Tothova et al.	Cohesin	Acute myeloid leukemia	^100^
2021	Atkin et al.	Cohesin	Acute myeloid leukemia	^101^
2020	Zeret	Altered chromatin loops associated with FOXA1 and GATA3	Breast cancer	^48^
2020	Voronina et al.	Chromothripsis	Various	^56^
2020	Li et al.	Somatic SVs	Various	^11^
2020	Akdemir et al.	Somatic SVs	Various	^60^
2020	Ooi et al.	TAD disruption	Gastric adenocarcinoma	^70^
2019	Yang et al.	TAD disruption	Leukemia	^69^
2019	Abdalla et al.	TAD disruption	Breast cancer	^71^
2019	Ma et al.	Cohesin-associated proteins (PDS5B and miRNA-223)	Pancreatic cancer	^99^
2018	Baxter et al.	Altered chromatin loops associated with FOXA1 and GATA3	Breast cancer	^47^

### Chromatin loops

Chromatin loops, identified in Hi-C data as concentrated regions with increased interactions^33,34^, play a crucial role in gene regulation. Whereas traditional cis-chromatin sequencing methods, such as Hi-C, can detect long-range chromatin loops, newer techniques, such as HiChIP^22,35^, TrAC looping^36^, and Capture-HiC^37^^,^ focus on the high-resolution sequencing of shorter-range loops. These methods provide PET images that aid in defining chromatin interactions, including enhancer–promoter interactions.

Incorporating the micrococcal nuclease enzyme into sequencing increases the resolution, which has led to recent methods, such as Micro-C^38^ and Microcapture-C^39^. These approaches offer high-resolution chromatin interaction data, enabling the examination of short-range chromatin interactions in human cancers.

These high-resolution interactions also provide insights into transcription factor (TF) binding within chromatin loops, offering new ways to study TF-driven childhood malignancies, such as rhabdomyosarcoma^40^ and Ewing sarcoma^41^. In rhabdomyosarcoma, fusion-positive subtypes show unique chromatin looping driven by the PAX3–FOXO1 oncoprotein^40,42^, offering a potential target for clinical interventions^35,43^. Fusion-negative subtypes exhibit different chromatin looping patterns, which can be influenced by clinical agents, such as trametinib^42,44,45^.

TF motifs play a role in shaping regulatory chromatin loops in human cancers. Recent studies have highlighted the role of noncoding genetic variants in altering chromatin interactions^46^ and emphasized the importance of pioneering factors, such as FOXA1 and GATA3, which are associated with altered chromatin loops in breast^47,48^ and bladder cancer^49^. Mutations in the CTCF-binding sites in various cancers also affect chromatin loop structures^50^.

In summary, the study of chromatin loops using advanced techniques offers insights into gene regulation and high-resolution interactions and their relevance to various diseases, including pediatric and adult cancers. These findings revealed potential targets for clinical intervention, highlighted the impact of genetic variants on chromatin interactions, and emphasized the role of pioneering factors and CTCF mutations in shaping chromatin loops in cancer.

### Structural variations

Recent studies have highlighted the power of Hi-C, particularly when combined with whole-genome sequencing, to identify SVs in the human genome, particularly in the context of cancer^10,51^. Hi-C, a chromatin sequencing and imaging method, has proven to be efficient for the de novo detection of SVs in cancer genomes^52^. These studies have several implications.

One key aspect is the newfound understanding of how SVs, such as deletions, inversions, and translocations, systematically impact enhancers and insulators that play crucial roles in gene regulation. Previous studies provide valuable insights into the relationship between gene regulation and SVs, shedding light on how SVs alter the regulatory functions of these elements.

The topological context of copy number variations (CNVs) and gene fusion events in cancer can also be revealed using Hi-C^49,53^. This revealed how these genetic alterations fit within chromatin domains. Furthermore, elucidating the effects of SVs on both noncoding and coding regions of the genome continue to provide insights into the epigenetic mechanisms that drive tumor development.

A notable study of leukemia genomes illustrated the potential of Hi-C for understanding SVs. In this case, SVs were found to modify the proximity of the *BCL11B* gene locus and its enhancer, thereby affecting gene expression in progenitor cells^54^. This study employed HiChIP data mapped onto patient-specific reference genomes to account for SVs, highlighting the importance of accounting for these variations in genome analysis.

The potential of Hi-C to reveal SV patterns, such as chromothripsis (multiple catastrophic chromosomal rearrangements), makes it a powerful tool, particularly in cases where other methods, such as SNP arrays or RNA-seq, may not capture these patterns effectively^55,56^.

Hi-C has been used to elucidate how SV-induced enhancer transpositions regulate the expression of oncogenic drivers^49,54,57–59^ in various cancer types, including leukemia, lymphoma, bladder cancer, adenocarcinoma, and pancreatic cancer^49,54,57–59^. These findings emphasize the potential of using low-depth Hi-C or Hi-ChIP to investigate patterns of SV and altered enhancer function in both clinical and basic research contexts. Ultimately, the integration of Hi-C with other sequencing methods offers a comprehensive view of the SVs in cancer genomes, providing valuable insights into the mechanisms driving cancer development and progression.

Complex SVs in human cancers often differ from those in normal tissues, and their association with chromatin interactions is becoming increasingly evident. There are common “SV pathways” leading to recurrent fusion-oncogene events, along with unique SV patterns associated with specific cancer types^11,60^. Specific types of cancer exhibit recurrent alterations in chromatin domain boundaries, connecting SVs with gene dysregulation. The integration of SVs into the context of transcriptional regulatory elements and domains is expected to define the structural drivers of cancer.

Overall, the combination of Hi-C and whole-genome sequencing is a powerful tool for understanding the role of SVs in cancer development and revealing their complex effects on the regulatory and structural architecture of the genome.

### TADs

A TAD is a region in the genome characterized by increased self-interactions^61,62^. These are crucial for maintaining proper genomic organization and function. TAD boundaries are primarily regulated by CTCF.

TADs are established during embryonic development^63,64^ and play a role in various cellular processes, including cell cycle regulation and DNA replication^25,26,65–67^. The dysregulation of TADs has been linked to diseases, particularly developmental malformations and tumorigenesis. De novo TAD formation, often referred to as neo-TAD, is a phenomenon in which SVs disrupt existing TADs in the genome^52^. This disruption creates new TAD boundaries, leading to changes in gene regulation. In the context of cancer research, neo-TAD formation is particularly relevant because it can result in the activation of oncogenes or the inactivation of tumor suppressor genes, contributing to tumorigenesis. Researchers have identified neo-TADs using Hi-C, and understanding their functional consequences is crucial for deciphering disease mechanisms^68^. Various studies have shown that TAD disruption can contribute to the development of cancers, such as leukemia^69^, gastric adenocarcinoma^70^, breast cancer^71^, and multiple myeloma^72^.

The strength of CTCF-mediated transcriptional insulation depends on factors such as the number of CTCF protein-binding sites, surrounding DNA sequences, and the location of these sites within or outside the TAD boundaries^7^. Targeting CTCF and TAD regulation may have broad applications in the treatment of cancer and other inherited diseases.

Recent studies have revealed subdomains within TADs known as chromatin nanodomains (CNDs)^73^, which persist in cells even when CTCF or cohesin, another important protein, is depleted, indicating their structural importance^74^. Understanding the role of CNDs, particularly in the context of enhancer–promoter interactions, can provide new insights into cancer development and progression.

Exploring the characteristics of self-renewing cancer stem cells, as well as the dynamic chromatin changes that occur during cancer progression using high-resolution techniques will be vital for advancing our understanding of cancer biology and treatment.

### CTCF

CTCF, a highly conserved protein, plays a central role in regulating 3D chromatin architecture and gene expression^61,75^. It is a versatile factor involved in processes such as transcriptional regulation, insulation, genomic imprinting, DNA repair and alternative splicing^76–78^. Its binding sites can be influenced by epigenetic modifications, mutations, and environmental factors, highlighting its dynamic nature^76^.

CTCF-mediated looping is highly conserved and critical during early embryonic development^79^. It regulates cell differentiation, fate, and development and is indispensable for these processes^80^. CTCF binding also responds to environmental stimuli^81^, further highlighting its dynamic role.

Recently, extensive research has focused on the association between CTCF and cancer. Studies have shown that CTCF aberrations can lead to tumorigenesis. For instance, in gliomas, mutations can lead to DNA hypermethylation, disruption of TAD boundaries, and oncogene activation^82^. In prostate cancer, CTCF interacts with other proteins to regulate gene expression^83^. Breast cancer metastasis can be controlled by CTCF and early growth response 1 (EGR1)^84^. Additionally, CTCF binding has been linked to UV damage and mutation hotspots in melanoma^85^.

The role of CTCF in human developmental diseases has also been discussed, with particular emphasis on its impact on cis-regulatory interactions and 3D chromatin architecture. Furthermore, the involvement of CTCF in cell aging and senescence has been highlighted, suggesting its potential role in restoring the function of senescent cells and its relevance to tumorigenesis^86,87^.

In summary, CTCF is a multifaceted protein with a wide range of functions, and its dysregulation can contribute to various diseases, particularly cancer and developmental disorders. Understanding the dynamic nature of CTCF and its role in these processes is essential for advancing our knowledge of disease mechanisms and potential therapeutic interventions.

### Cohesin

Cohesin is a well-known structural maintenance protein in the chromosome complex that plays a pivotal role in sister chromatid cohesion during the cell cycle, ensuring proper chromosome segregation^88^. It also contributes to maintaining the 3D architecture of interphase chromosomes^8,33,89^. After the establishment of CTCF anchors, cohesin assists in the loop extrusion process, which can occur through various mechanisms, including diffusion^90^, ATP hydrolysis^91^, and translocating factors, such as RNA polymerase II^92^.

This protein complex plays a regulatory role in gene expression by modulating chromatin structure. It collaborates with CTCF and other proteins to regulate gene expression and genome organization. Cohesin and CTCF work together to separate chromatin into distinct spatial domains, facilitating long-range interactions and coregulating genes within those domains^9^.

In the context of cancer, dysregulation of cohesin-related proteins is associated with tumor initiation and development^93,94^. Mutations in genes encoding cohesin proteins have been identified in various cancers, particularly myeloid malignancies^95^ and solid tumors^96,97^. The hinge domain of the cohesin complex plays a critical role in these mutations^98^. Additionally, cohesin-associated proteins, such as PDS5B and miRNA-223, have been linked to cancer, suggesting that these proteins may be potential therapeutic targets^99^.

Targeting dysfunctional or mutated cohesin complexes may be new strategies for cancer therapy, and patients with cohesin mutations can be considered distinct subgroups in clinical trials. Abnormal expression of cohesin complex genes can also serve as a prognostic tool in cancer.

In hematological malignancies, cohesin mutations are key drivers of DNA damage repair and alterations in chromatin architecture, thereby providing therapeutic opportunities^100^. DNA breakage at the CTCF/cohesin binding sites may become a robust early detection tool for individuals susceptible to blood cancer^101^.

Interactions of the cohesin complex with other architectural proteins such as CTCF and polycomb group proteins emphasize its fundamental role in 3D chromatin structure and gene regulation^102^. Understanding the differences between cohesin subunits such as STAG1 and STAG2 could lead to more targeted therapeutic approaches.

In conclusion, the cohesin protein complex is a versatile player in various biological processes and has the potential to open new therapeutic avenues for cancer and other diseases, especially as advanced technologies provide deeper insights into its functions.

## Detection of structural variants that drive oncogene activation

Hi-C has emerged as a powerful tool for identifying cancer-specific alterations within TADs, shedding light on their pivotal role in cancer development^60,75^. These alterations originate primarily from SVs and include duplications, deletions, inversions, and translocations. These genomic rearrangements have the potential to disrupt the 3D organization of the genome^52^. Such disruption, often referred to as enhancer hijacking, is known to activate oncogenes, promoting uncontrolled cell proliferation and tumor growth, and has been documented across a spectrum of cancers, including acute myeloid leukemia^2,103^, neuroblastoma^104^, medulloblastoma^105^ and colorectal cancer^106^.

**Fig. 3:**
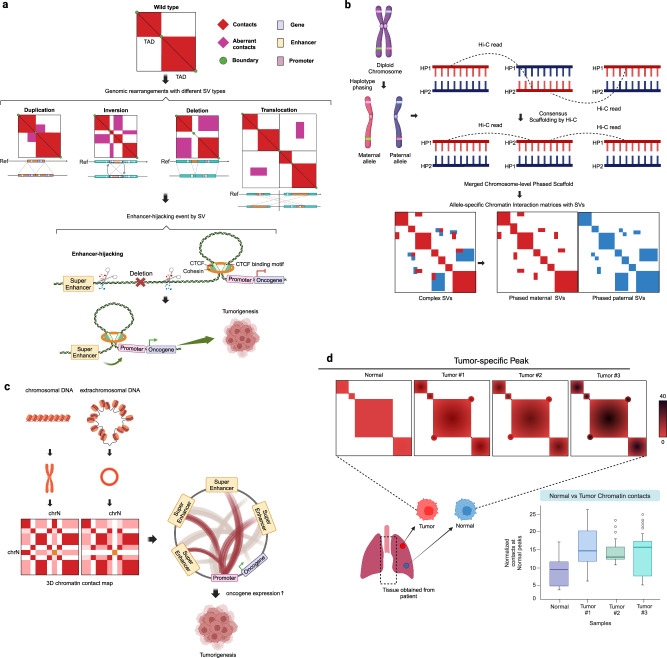
Applications of Hi-C in human cancer genome research. **a** Hi-C contact matrices can be used to effectively identify enhancer hijacking events resulting from SVs. These matrices can reveal alterations in enhancer–promoter interactions resulting from genomic rearrangements. Enhancer hijacking occurs when regulatory elements such as super-enhancers are misdirected and interact with the promoters of genes, including oncogenes, with which they normally do not interact. This aberrant interaction can lead to dysregulated gene expression, contributing to cancer oncogenesis and development. **b** Chromosome-level haplotype blocks are constructed using Hi-C reads to link alleles in contigs, thus revealing specific combinations of genetic variants present on each chromosome. The precise characterization of individual genomes, facilitated by chromosome-level haplotype blocks, allows for a better understanding of genetic diversity and inheritance patterns within populations. It provides information on the specific combinations of genetic variants present on each chromosome, aiding in personalized medicine and genetic studies. **c** In the context of ecDNA detection, Hi-C contact matrices leverage the characteristic pattern of ecDNA interactions, which typically involve local self-interactions and lack interchromosomal interactions. This distinctive behavior allows Hi-C to identify and characterize ecDNAs within the genomic landscape, particularly in the context of diseases, such as cancer. **d** Comparative analysis of cancer cell lines and normal mammary cells revealed CTCF-dependent changes in chromatin organization, affecting chromatin accessibility, transcription, and epigenetic features. These disruptions provide critical insights into the structural and functional aspects of 3D chromatin in cancer cells.

Superenhancers (SEs) can also be redirected by enhancer hijacking to promote the expression of oncogenes or other genes that drive tumorigenesis. This aberrant activation of SEs substantially contributes to cancer development and progression^107,108^ (Fig. 3a). Understanding how SEs are hijacked in cancer cells has become a crucial focus for both basic research and potential therapeutic strategies in the field of oncology.

Furthermore, Xu et al.^109^ harnessed Hi-C data derived from cancer cell lines and patient samples. Their work precisely identified specific loci where recurrent changes manifested within the 3D structure of the genome. Notably, these structural alterations are strongly associated with well-known oncogenes, such as *TERT, MYC*, and *CCND1*. These findings were substantiated by CRISPR-Cas9 genome engineering. This cutting-edge technology enables researchers to anticipate the activation of oncogenes by scrutinizing the interactions between distinct chromatin regions and SE activity.

Kim et al.^110^ revealed a pivotal insight: recurrent enhancer hijacking, orchestrated by SVs, can instigate a surge in oncogenic expression, all without a pronounced surge in CNVs. This discovery further underscores the indispensable role of Hi-C data in revealing the intricate web of oncogenic regulation in cancer, thereby shedding light on the multifaceted mechanisms driving cancer development and progression. This study adds a vital piece to the puzzle of cancer research by showing the complexity of cancer genomes and how seemingly subtle changes in their 3D structures can have profound consequences in the context of oncogenic expression.

## Reconstruction of complex genomic rearrangements at the haplotype level

Hi-C data play a pivotal role in haplotype phasing, a fundamental process that is crucial for identifying the parental origin of genetic variants located along a chromosome^12^. Haplotype phasing can be performed either statistically or experimentally. The statistical method relies on population genetic data and linkage disequilibrium but may introduce errors at recombination hotspots^110^. In contrast, experimental phasing uses multiple variants within sequencing reads but faces challenges owing to the limited prevalence of heterozygous polymorphisms^111^.

Using the Hi-C data, primary contigs are assembled and extended into chromosome-level allele-specific contigs. Primary contigs are partitioned into allele blocks, and linkages are established, ultimately leading to the creation of extended allele-specific contigs (Fig. 3b). Several computational algorithms, such as HapCUT^112^, leverage Hi-C data for this purpose.

Recent efforts have integrated Hi‒C data with other sequencing techniques, especially long-read sequencing, leading to substantial improvements in the fractionation of phased variants^12,113^. The synergy between Hi-C and long-read sequencing, linked-read sequencing, and genome assembly methods can achieve more comprehensive and precise haplotype phasing. This method plays a crucial role in understanding complex genomic rearrangements at the haplotype level and offers valuable insights into allele-specific chromatin interactions, particularly in cancer research. It not only aids in the identification of allele-specific structural variants and somatic mutations but also provides a nuanced perspective on somatic clonal evolution^114^. Analysis of allele-specific somatic mutations and variants enables deeper exploration of the dynamics of genomic changes in the context of disease progression, considerably enhancing our understanding of these processes, particularly in the study of diseases, such as cancer.

Notably, Garg et al.^115^ developed an efficient approach for chromosome-level haplotype reconstruction using Hi-C and long-read sequencing in cancer cell lines, surpassing the existing methods in terms of accuracy and speed. This approach revealed a detailed structural variant landscape, which not only aids in personalized diagnosis but can also predict therapeutic responses in patients with cancer.

In summary, integrating Hi-C data with advanced sequencing techniques is crucial for haplotype phasing and offers insights into complex genomic rearrangements, allele-specific interactions, and somatic evolution.

## EcDNA detection

EcDNA refers to circular DNA segments that exist outside linear chromosomes and vary in size from kb to Mb. EcDNA plays a crucial role in generating tumor heterogeneity, contributing to drug resistance and poor prognosis^13^. It is considered to be a major factor in the genomic diversity of cancers, particularly glioblastoma^116,117^.

The identification of ecDNA has been substantially facilitated by the emergence of Hi-C technology. This is primarily due to unique characteristics that distinguish ecDNA from conventional chromosomal DNA^53^. These characteristics encompass a range of distinctive features, including specific spatial proximities, the formation of chimeric DNA molecules, unconventional DNA interactions, SVs, and enhanced signals detectable within the Hi-C data. This technology effectively leverages these traits to accurately locate and differentiate ecDNA within the genomic landscape.

The principal objective of Hi-C is to reveal the intricate spatial organization of various genomic regions within the cell nucleus^75^. Notably, ecDNA exhibits distinct spatial relationships compared with chromosomal DNA^13,118^, making Hi-C exceptionally adept at capturing these spatial proximities. During Hi-C, DNA fragments originate from diverse genomic amalgamation areas, resulting in the formation of chimeric DNA molecules^75^. The amalgamation of ecDNA into chimeric structures results in recognizable patterns that serve as unequivocal indicators of the presence of ecDNA.

Specifically, regions containing ecDNA may exhibit higher concentrations of Hi-C interactions^13,118^ and coamplify enhancer elements^119,120^, signifying areas of elevated spatial interactions (Fig. 3c). These intensified signals in the Hi-C data further enhanced the detectability of ecDNA within the genome. By harnessing these unique attributes, Hi-C offers a robust and comprehensive approach for detecting and characterizing ecDNA, making a substantial contribution to our understanding of the genomic landscape. This is particularly vital in the context of diseases, such as cancer, where ecDNA functions as a mobile enhancer that amplifies chromosomal transcription.

## Characterization of 3D chromatin organization in cancer through comparative analysis

Chromatin exhibits a 3D architecture that plays essential roles in fundamental biological processes, such as the cell cycle, DNA replication, cellular differentiation, and transcription regulation^121^. Conversely, disruptions in these 3D structures are closely associated with the emergence of anomalies and various diseases, among which cancer is particularly notable^3^. The association between disruptions in 3D chromatin organization and cancer is an area of active research with substantial implications for our understanding of cancer development and progression.

Kim et al.^122^ comprehensively characterized 3D chromatin organization in various breast cancer cell lines and compared it to that in normal mammary epithelial cells and tissues (Fig. 3d). This study revealed that CTCF-dependent cancer-specific changes in 3D chromatin organization are associated with perturbed chromatin accessibility and transcriptional dysregulation, affecting compartment domains, TADs, and chromatin loops. These changes, which are strongly linked to epigenetic modifications and gene expression, are partially conserved in cancer tissues, highlighting the disruptive nature of 3D chromatin organization in breast cancer.

In summary, disruptions in 3D chromatin organization in cancer revealed a connection between these structural changes, epigenetic features, and gene expression, offering critical insights into the 3D chromatin structural features of cancer. In addition to previous findings, studies have shown that drug resistance or drug efficacy causes differences in 3D chromatin organization^123,124^. Understanding how drugs affect chromatin organization can provide insight into the mechanisms underlying drug resistance and efficacy, potentially leading to the development of more effective treatments. This knowledge serves as a foundation for diagnostic and therapeutic investigations and advances our understanding of cancer etiology.

## Concluding remarks

Here, we review how 3C technology has revolutionized our understanding of cancer biology by revealing the complexities of 3D chromatin interactions, including chromatin loops, TADs, and interactions between regulatory elements and oncogenes. This knowledge offers promising avenues for identifying critical factors involved in tumorigenesis and potential therapeutic interventions.

3C-based techniques, such as HiChIP and Micro-C, can provide higher-resolution data, which are particularly valuable for studying enhancer–promoter interactions and understanding the impact of oncogenic drivers on gene expression in cancer. 3C technology has also emerged as an effective tool for detecting SVs within cancer genomes, shedding light on how these alterations disrupt enhancers and insulators and affect both coding and noncoding regions.

The process of haplotype phasing, which is vital for studying oncogene activation, has been streamlined using Hi-C technology, particularly when integrated with long-read sequencing. This integration enables precise identification of allele-specific SVs and somatic mutations. The unique ability of Hi-C-based methods to detect ecDNA is essential for understanding its role in cancer development, as it often acts as a mobile enhancer and amplifies gene transcription. Comparative analyses of 3D chromatin organization in cancer have revealed disruptions associated with epigenetic and gene expression alterations, providing insights into the importance of 3D chromatin structures in cancer development.

Ongoing advancements, including the integration of multiple omics data and the development of single-cell multiomics 3C techniques, offer opportunities to characterize cell type-specific chromatin organization in complex tissues and heterogeneous cancer genomes. Despite this progress, challenges remain, such as handling repetitive sequences and refining methods for mapping structural variants.

In summary, 3C technology has substantially advanced our understanding of cancer biology, offering exciting possibilities for more targeted therapies and shaping our knowledge of cancer biology and its clinical applications.
