# Author Correction: 3C methods in cancer research: recent advances and future prospects

**DOI:** 10.1038/s12276-024-01349-1

**Published:** 2024-11-21

**Authors:** Insoo Yoon, Uijin Kim, Kyung Oh Jung, Yousuk Song, Taesoo Park, Dong-Sung Lee

**Affiliations:** 1 Department of Life Science, University of Seoul, Seoul, Republic of Korea; 2 Department of Anatomy, College of Medicine, Chung-Ang University, Seoul, Republic of Korea

**Keywords:** Cancer genomics, Epigenomics

Correction to**:** *Experimental & Molecular Medicine* 10.1038/s12276-024-01236-9, published online 25 April 2024

Upon further review, it has come to our attention that Dr. Kyung Oh Jung was inadvertently omitted from the original author list. Dr. Jung contributed significantly to the conception, design, data analysis, and manuscript preparation of the article. Accordingly, Dr. Jung has now been included as the first author of the article. The revised author list is as follows: Insoo Yoon^1^, Uijin Kim^1^, Kyung Oh Jung^2^, Yousuk Song^1^, Taesoo Park^1^, & Dong-Sung Lee^1^

^1^Department of Life Science, University of Seoul, Seoul, 02504, Republic of Korea

^2^Department of Anatomy, College of Medicine, Chung-Ang University, Seoul 06974, Republic of Korea

All authors have agreed to this correction and have confirmed Dr. Jung’s substantial contributions to the research and the manuscript.

Additionally, the acknowledgments section has been updated to include additional funding that supported this work. The correct funding statement is as follows:

“This work was supported by the 2022 Advanced Facility Fund of the University of Seoul.”

We apologize for any inconvenience caused by these omissions and appreciate the opportunity to correct the record. We remain committed to transparency and accuracy in our published work.

The original article has been corrected.

